# ﻿ *Pseudospermaarenarium* (Inocybaceae), a new poisonous species from Eurasia, based on morphological, ecological, molecular and biochemical evidence

**DOI:** 10.3897/mycokeys.92.86277

**Published:** 2022-08-30

**Authors:** Ya-Ya Yan, Yi-Zhe Zhang, Jukka Vauras, Li-Na Zhao, Yu-Guang Fan, Hai-Jiao Li, Fei Xu

**Affiliations:** 1 School of Public Health and Management, Ningxia Key Laboratory of Environmental Factors and Chronic Diseases Control, Ningxia Medical University, Yinchuan, Ningxia, 750004, China Ningxia Medical University Yinchuan China; 2 National Institute of Occupational Health and Poison Control, Chinese Centre for Disease Control and Prevention, Beijing, 100050, China National Institute of Occupational Health and Poison Control, Chinese Centre for Disease Control and Prevention Beijing China; 3 Key Laboratory of Tropical Translational Medicine of Ministry of Education, Hainan Key Laboratory for R & D of Tropical Herbs, School of Pharmacy, Hainan Medical University, Haikou, Hainan, 571199, China University of Turku Turku Finland; 4 Biological Collections of Åbo Akademi University, Herbarium, Biodiversity Unit, University of Turku, FI-20014 Turku, Finland Hainan Medical University Hainan China; 5 Physical and Chemical Department, Ningxia Hui Autonomous Region Center for Disease Control and Prevention, Yinchuan, Ningxia, 750004, China Physical and Chemical Department, Ningxia Hui Autonomous Region Center for Disease Control and Prevention Yinchuan China

**Keywords:** Agaricales, muscarine, mushroom toxin, new taxon, poisonous mushroom, ultra-high performance liquid chromatography-tandem mass spectrometry

## Abstract

In this study, *Pseudospermaarenarium* is proposed as a new species, based on morphological, ecological, molecular and biochemical evidence. The new species grows on sandy ground under *Populus* and *Pinussylvestris* in north-western China and northern Europe, respectively. It is characterised by the combination of the robust habit, nearly glabrous pileus, large cylindrical basidiospores, thin-walled cheilocystidia and ecological associations with *Populusalba* × *P.berolinensis* and *Pinussylvestris* and unique phylogenetic placement. Additionally, a comprehensive toxin determination of the new species using ultra-high performance liquid chromatography-tandem mass spectrometry was conducted. Results showed that it was a muscarine-positive species. The content were approximately five times higher in the pilei [4012.2 ± 803.1–4302.3 ± 863.2 mg/kg (*k* = 2, *p* = 95%)] than in the stipes [850.4 ± 171.1–929.1 ± 184.2 mg/kg (*k* = 2, *p* = 95%)], demonstrating the severity of mushroom poisoning when patients consumed different parts of the poisonous mushroom. Amatoxins, phallotoxins, ibotenic acid, muscimol, psilocybin and psilocin were not detected.

## ﻿Introduction

Inocybaceae is a family of agarics that contains many poisonous species. However, Kosentka et al. (2013) found that the most recent common ancestor of the family did not contain muscarine. Recognising its species diversity and detecting its toxins are essential to control and prevent poisoning incidents (Li et al. 2020; Deng et al. 2021a). According to the latest molecular phylogeny, seven genera were treated in Inocybaceae (Matheny et al. 2020). *Pseudosperma*, referred to as Inocybesect.Rimosae sensu stricto (Larsson et al. 2009) or Pseudosperma clade (Matheny 2009), is one of the muscarine-containing genera in the family with numerous cryptic and semi-cryptic species. It is characterised by rimulose to rimose pileus, furfuraceous to appressed furfuraceous stipe with flocculose apex, elliptic to sub-phaseoliform basidiospores, the absence of pleurocystidia and the presence of thin-walled cheilocystidia. Ninety-seven *Pseudosperma* taxa have been recorded in the IndexFungorum database (www.indexfungroum.org; retrieved 7 May 2022). Of these, more than 40 taxa have been reported or originally described in Europe (Bandini and Oertel 2020). Since the establishment of the genus in 2020, 16 new taxa have been discovered in Asia and Europe in the past 2 years alone (Bandini and Oertel 2020; Cervini et al. 2020; Jabeen and Khalid 2020; Saba et al. 2020; Yu et al. 2020; Bandini et al. 2021; Jabeen et al. 2021). However, the species diversity of *Pseudosperma* is still poorly explored in East Asia. In China, only six taxa have been verified, including three recently described species, viz., *P.yunnanense*, *P.neoumbrinellum* and *P.citrinostipes* (Bau and Fan 2018; Yu et al. 2020).

Ecologically, *Pseudosperma* species have an ectomycorrhizal symbiosis with various plants and are commonly found in north temperate forests dominated by *Betula*, *Cedrus*, *Populus*, *Pinus*, *Picea*, *Quercus*, *Salix* etc. During field surveys in north-western China, a poisonous Inocybaceae mushroom collected under *Populus* plantations caught the authors’ attention because of its strikingly robust habit. This stout Inocybaceae species has led to three poisoning incidents, with a total of seven patients in north-western China during the past 2 years. Two of these occurred in September in Ningxia and Shanxi in 2020 and another occurred in Ningxia in October 2021 (Li et al. 2021a, 2022). All patients from the three poisoning incidents suffered from classic parasympathetic nervous system stimulation syndromes. After microscopic examinations and molecular analyses, mushroom specimens obtained from poisoning locales, together with a European specimen, were proven as a new *Pseudosperma* species. Discussions on the distribution, relationships and distinction of the new species and its affinities are also provided. Additionally, to better understand the toxicity of the new species and contribute to their poisoning control and prevention, 11 major mushroom toxins, namely, two isoxazole derivatives (ibotenic acid and muscimol), two tryptamine alkaloids (psilocybin and psilocin), three amatoxins (α-, β- and γ-amanitin), three phallotoxins (phalloidin, phallacidin and phallisacin) and muscarine, were assayed.

## ﻿Methods

### ﻿Sampling, morphological observations and descriptions

The Chinese materials were collected in sandy poplar plantations from Ningxia Hui Autonomous Region and Shaanxi Province, where there is a temperate continental climate. The European material JV26578 was collected in a seashore forest from Estonia, in a hemiboreal zone. Macroscopic features were described, based on fresh materials and colour photographs. A small piece of the pileus, lamella or stipe tissue was mounted in 5% aqueous potassium hydroxide (KOH) on the slide and then examined using a light microscope when the tissue was completely rehydrated. Microscopic structures, including basidiospores, basidia, cheilocystidia, hymenophoral trama, caulocystidia, pileipellis and stipitipellis, were examined from rehydrated materials. The measurements of micro-structures follow Fan and Bau (2013) and Yu et al. (2020). The number of measured basidiospores is given as an abbreviation [n/m/p], which denotes n spores measured from m basidiomata of p collections. The measurements and Q values are given as (a)b–c(d), “b–c” covers a minimum of 90% of the measured values, “a” and “d” represent the extreme values; Q means the ratio of length/width in an individual basidiospore, Q_m_ is the average Q of all basidiospores ± sample standard deviation (Ge et al. 2021; Na et al. 2022). Colour designations follow Kornerup and Wanscher (1978). Voucher specimens were deposited in the Herbarium of Herbarium of Changbai Mountain Nature Reserve (ANTU) with FCAS numbers and TUR-A.

### ﻿DNA extraction, polymerase chain reaction, sequence amplification and data analysis

Genomic DNA was extracted from silica-dried materials using the NuClean Plant Genomic DNA Kit (ComWin Biotech, Beijing). The internal transcribed spacer (ITS) region, the nuclear large subunit (nLSU) and the RNA polymerase II second largest subunit (RPB2) sequences were amplified and sequenced separately by using primer pairs ITS1F/ITS4 (Gardes and Bruns 1993), LR0R/LR7 (Vilgalys and Hester 1990) and RPB2-6F/RPB2-7.1R (Matheny 2005). The PCR thermocycling protocol was 95 °C for 1 min at first, then followed by 35 cycles of denaturation at 95 °C for 30 s, annealing at 52 °C for 1 min, extension at 72 °C for 1 min and a final extension at 72 °C for 8 min (Wang et al. 2021). Sequencing work was done by Sangon Biotech (Shanghai) Co., Ltd. Sequences of related taxa in *Pseudosperma*, retrieved from previous studies, were downloaded from GenBank (https://www.ncbi.nlm.nih.gov/) for phylogenetic analysis (Suppl. material 1). *Mallocybeterrigena* (Fr.) Matheny, Vizzini & Esteve-Rav. was used for the outgroup. The sequence data matrix for each locus was aligned by Mafft online service (https://mafft.cbrc.jp/alignment/server/) (Katoh et al. 2019) and manually adjusted by BioEdit 7.0.9.0 (Hall 1999). The aligned datasets were combined with Mega 5.02 (Tamura et al. 2011). MrModeltest v.2.3 was used to determine the optimal substitution model for each locus with the Akaike Information Criterion (Nylander 2004). Bayesian Inference (BI) analyses, executed in MrBayes v.3.2.7a (Ronquist et al. 2012), were run for 1,235,000 generations using four Metropolis-Coupled Monte Carlo Markov chains to calculate posterior probabilities and the standard deviation of the split frequencies was terminated at 0.009977. Maximum Likelihood (ML) analysis was conducted in W-IQ-TREE Web Service (http://iqtree.cibiv.univie.ac.at/) with 1,000 replicates (Trifinopoulos et al. 2016).

### ﻿Toxin detection

Ultra-high performance liquid chromatography-tandem mass spectrometry (UPLC-MS/MS) was performed for toxin detection. Detailed mushroom sample preparations, analysis of muscarine, amatoxins and phallotoxins (Alta Scientific Co., Ltd., Tianjin, China) referred to our previous works (Xu et al. 2020a, 2020b).

Detailed information for analysis of ibotenic acid and muscimol (Alta Scientific Co., Ltd., Tianjin, China) are as follows: chromatographic separation was conducted on an ACQUITY UPLC C8 column (2.1 × 100 mm, 1.7 μm; Waters, USA). Acetonitrile (A) and 4% formic acid aqueous solution (B) were used as mobile phase solvent flowing at 0.3 ml/min. The column was eluted by 2% A for 1.0 min, followed by 2%–70% A for 1.0 min, then by 70% A for 1.0 min and then by 70%–2% A for 0.5 min, finally by 2% A for 1.5 min. The analytical column was set at 40 °C. The injection volume was 10 μl. The positive MS/MS conditions can refer to muscarine (Xu et al. 2020b). The ion pairs were 115.1 > 68.1 (Cone at 16 V; Collision at 12 V), 159.1 > 113.1 (Cone at 16 V; Collision at 12 V) for ibotenic acid, as well as 115.1 > 98.1 (Cone at 15 V; Collision at 10 V), 115.1 > 68.1 (Cone at 15 V; Collision at 18 V) for muscimol.

For the analysis of psilocybin and psilocin (Alta Scientific Co., Ltd., Tianjin, China), the detailed descriptions are as follows. ACQUITY UPLC T3 column (2.1 × 100 mm, 1.7 μm; Waters, USA) was used as the separation column. The mobile phases were acetonitrile (A) and 10 mmol/l ammonium acetate aqueous solution (B). The flow rate was 0.3 ml/min. The column was eluted by 0% A for 0.5 min, followed by 0%–85% A for 4 min, then by 85% A for 1.5 min and then by 85%–0% A for 1.5 min, finally by 0% A for 2 min. The analytical column was set at 40 °C and the injection volume was 10 μl. The positive MS/MS conditions can refer to muscarine (Xu et al. 2020b). The ion pairs were 285.1 > 85.2 (Cone at 16 V; Collision at 18 V), 285.1 > 240.1 (Cone at 16 V; Collision at 17 V) for psilocybin and 205.1 > 58.2 (Cone at 26 V; Collision at 13 V), 205.1 > 160.1 (Cone at 26 V; Collision at 13 V) for psilocin.

## ﻿Results

### ﻿Phylogenetic analyses

**Figure 1.**
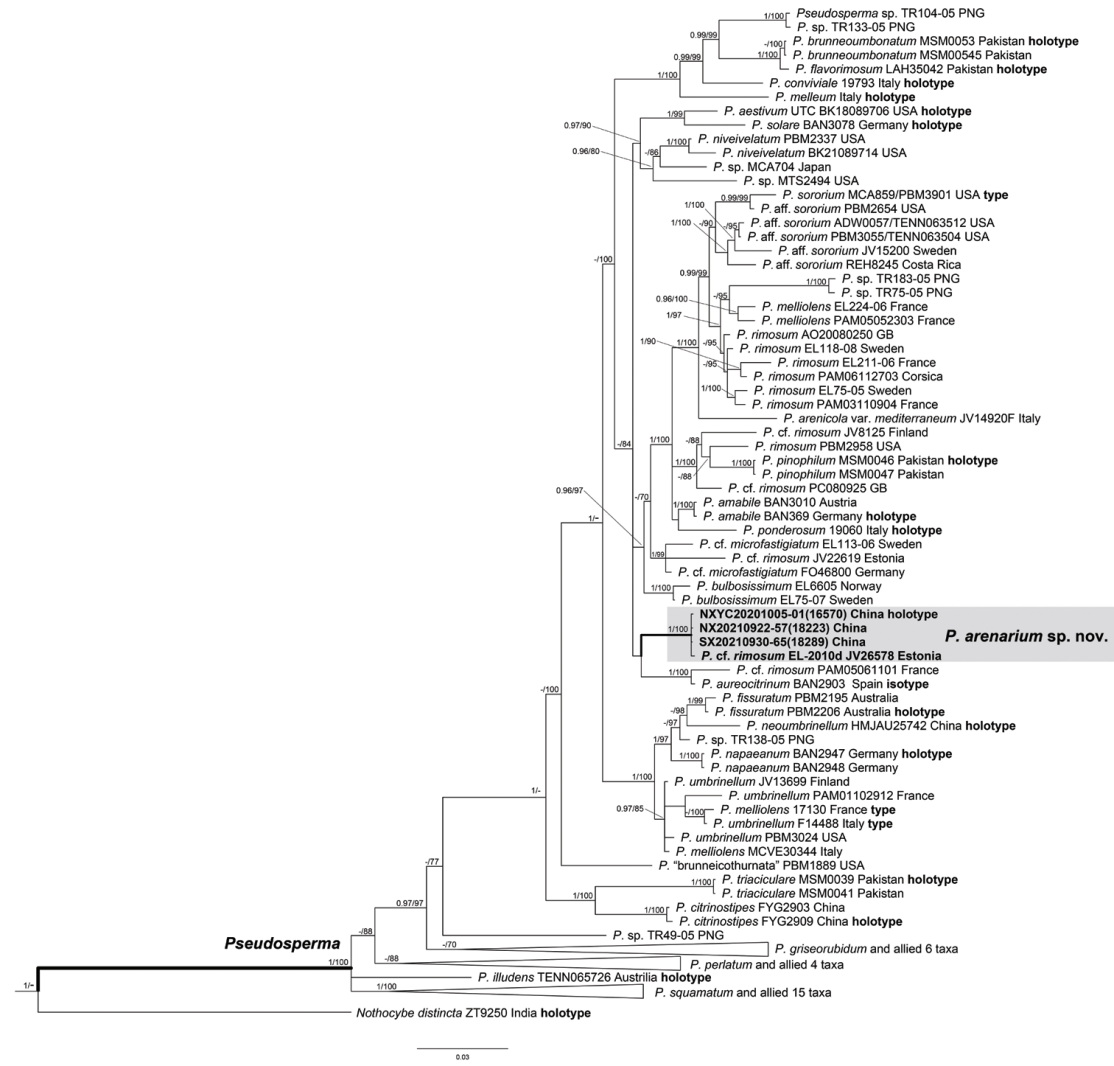
Phylogram generated by Bayes Inference (BI) analysis, based on a combined sequences dataset from nuclear genes (rDNA-ITS, nrLSU and *rpb2*), rooted with *Mallocybeterrigena* (Pruned). Bayesian Inference posterior probabilities (BI-PP) ≥ 0.95 and ML bootstrap proportions (ML-BP) ≥ 70 are represented as BI-PP/ML-BP.

Nine sequences (three ITS, three LSU and three *rpb2*) were newly generated and submitted to GenBank. The best-fit model selected by MrModeltest was GTR+I+G for each gene equally. The three-gene data matrix consisted of 104 taxa and 2890 sites. The final multilocus alignment used for phylogenetic reconstruction was submitted to TreeBase (ID29310). The Bayesian Inference and Maximum Likelihood trees were similar in topology; thus, only the BI tree was presented (Fig. 1). In the BI tree (Fig. 1), all *Pseudosperma* taxa grouped in a fully supported clade and three Chinese specimens and a European specimen (JV26578) clustered in an independent lineage with full support. The lineage clustered with the lineage composed of *I.aureocitrinum* (Esteve-Rav.) Matheny & Esteve-Rav. but with limited support.

### ﻿Taxonomy

#### ﻿ Pseudosperma arenarium

Taxon classificationFungiAgaricales Inocybaceae

Y.G. Fan, Fei Xu, Hai J. Li & Vauras sp. nov.

2575C825-2C17-5A12-9CB6-ECD00C8718B9

 842603

Figs 2 , 3

##### Etymology.

refers to its habitat of sandy soils.

##### Holotype.

China, Ningxia Hui Autonomous Region, Wuzhong, Yanchi County, Yanchi Railway Station, on sandy ground under *Populusalba × P.berolinensis*, 5 Oct 2020, NXYC20201005-01 (FCAS3571, holotype). GenBank accession nos.: ITS-OM304278, LSU-OM304287, *rpb2*-OM421667.

**Figure 2.**
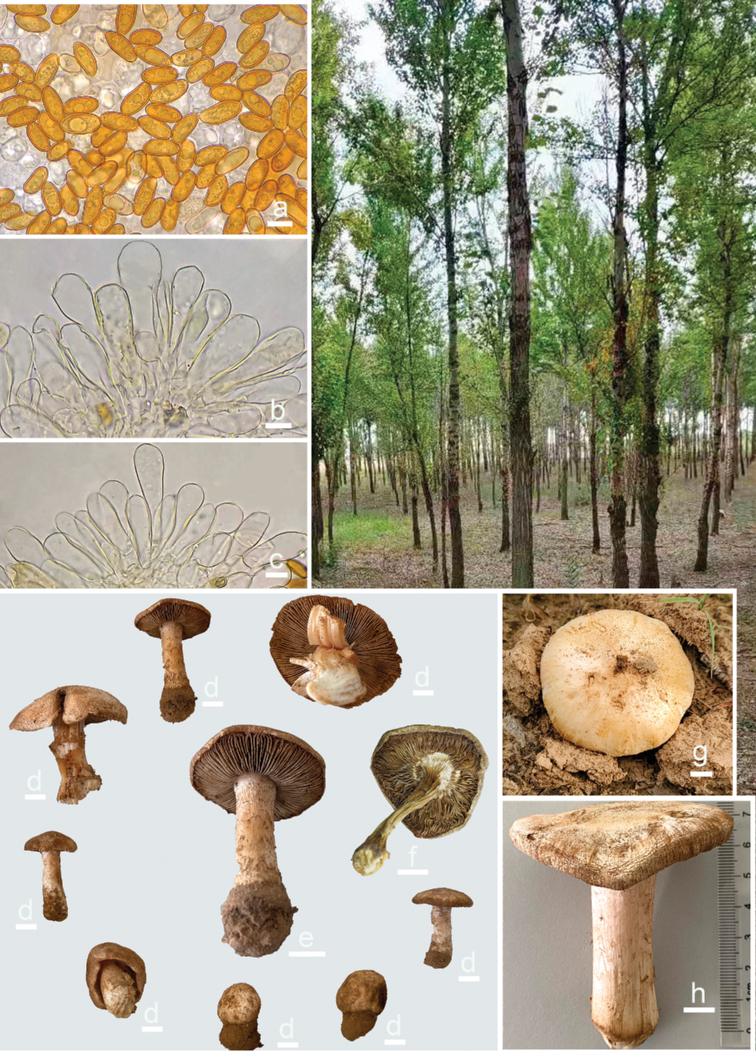
*Pseudospermaarenarium* and its habitat **a** basidiospores **b, c** cheilocystidia **d–h** basidiomata. Scale bars: 10 μm (**a–c**); 10 mm (**d–h**). Photos by Xu Fei, Li Hai-Jiao & Zhao Li-Na.

##### Diagnosis.

Basidiomata robust, pileus beige, ivory white or yellowish; basidiospores > 13 μm, cylindrical to cylindrical-ellipsoid, cheilocystidia thin-walled. Occurs under artificial plantations of *Populusalba × P.berolinensis* or open seashore forest of *Pinussylvestris* Linn. Differs from *P.arenicola* by longer basidiospores and phylogenetic distance.

**Figure 3.**
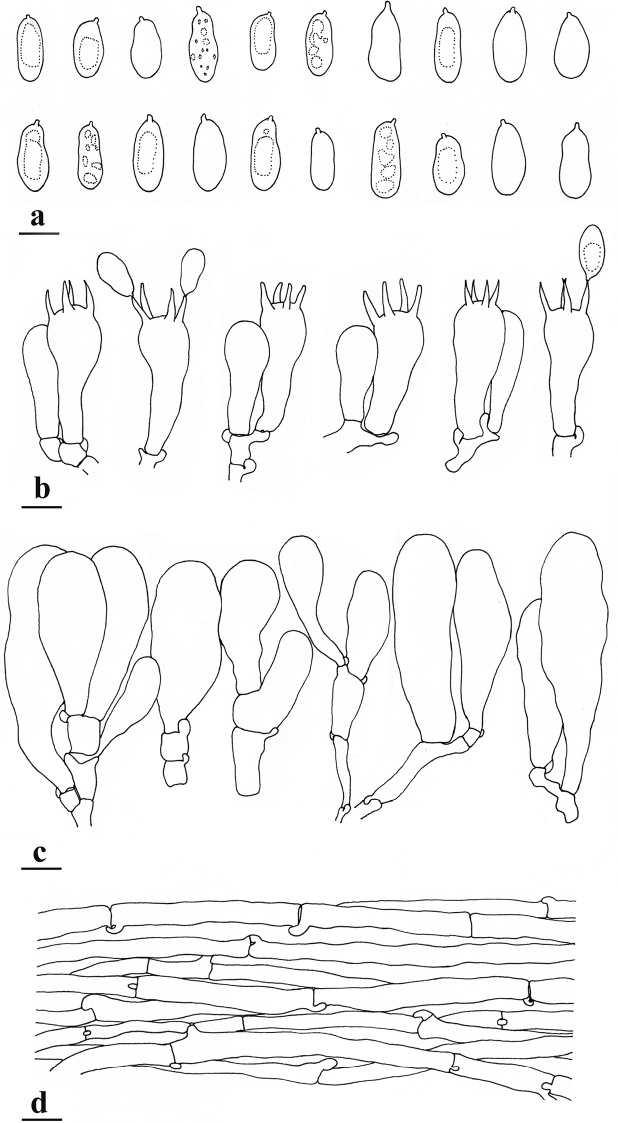
Microfeatures of *Pseudospermaarenarium* (holotype) **a** basidiospores **b** basidia **c** cheilocystidia **d** pileipellis. Scale bars: 10 μm (**a–d**). Line drawings by Li Hai-Jiao.

##### Basidiomata.

medium-sized, robust. Pileus 35–65 mm in diameter, spherical to hemispherical when young, convex, dome-shaped to applanate when mature, not umbonate, margin inrolled at first, becoming depressed, straight, to uplifted or recurved in age; surface dry, glabrous to slightly fibrillose, occasionally rimulose to rimose at the margin, with distinct sandy remnants; yellowish (1A2) to ochraceous (1A4), paler outwards, ivory white (1B1) to greyish-white (1B2) when dried. Lamellae crowded, up to 8 mm in width, adnexed to sub-free, not equal, alternately distributed with three tiers of lamellula, initially pure white to creamy white (1A2), becoming yellowish (4A3), brownish (5B6) to cinnamon (5C8) with age, yellowish-brown (4B8) to dark brown (6C7) after drying, edge pinkish-white, fimbricate. Stipe 40–100 × 7–20 mm, solid, equal or slightly tapering downwards, sometimes swollen towards the base, but not marginate, longitudinally fibrillose with scattered squamules, white to ivory white (1B1–1B2) with pinkish tinge (11A3) when fresh, yellowish (5A4) to brownish (5B5) upon drying. Context solid, white and fleshy in pileus, 2–5 mm in thickness, fibrillose in the stipe, striate and shiny, white to somewhat pinkish (7A2). Odour fungoid or slightly spermatic.

##### Basidiospores.

[170/6/4] (13–)14–20(–21) × (6–)7–9.2(–11) μm, median 16.4 × 7.8 μm, Q = (1.6–)1.75–2.64(–2.95), Q_m_ = 2.12 ± 0.27, yellow-brown, smooth, mostly cylindrical to cylindrical-ellipsoid, less often narrowly ellipsoid to nearly phaseoliform. Basidia 32–42 × 11–14 μm, clavate, usually narrower downwards, four-spored, sterigmata up to 10 μm long, translucent with oily inclusions, occasionally with yellowish pigments. Pleurocystidia absent. Lamellae edge sterile. Cheilocystidia 30–77 × 12–23 μm, thin-walled, colourless, broadly clavate or fusiform, rarely septate, translucent or occasionally with golden yellow inclusions, walls yellowish. Caulocystidia not observed. Hymenophoral trama nearly regularly arranged, composed of translucent and pale yellow, thin-walled hyphae up to 22 μm wide. Pileipellis a cutis, regularly arranged, orange-brown to brownish in 5% KOH, composed of cylindrical hyphae 4–15 μm in diameter; pileal trama made up of compact, parallel, hyaline hyphae, pale yellow in mass. Stipitipellis a cutis frequently disrupted by loose hyphal projections, hyphae thin-walled, colourless, 3–16 μm wide. Stipe trama regularly and densely arranged, yellowish in mass, hyphae thin-walled, colourless, 8–21 μm wide. Oleiferous hyphae 4–15 μm wide, present in pileus and stipe, bright yellow, smooth, often bent, occasionally branched or catenate. Clamp connections are common in all tissues.

##### Habitat.

individual or scattered on sandy and saline-alkali soil under artificial plantations of *Populusalba* × *P.berolinensis* in China and open seashore forest of *Pinussylvestris* in Estonia. Fruiting in autumn, from late September to early October.

##### Known distribution.

China (Ningxia and Shaanxi), Estonia.

##### Additional materials examined.

China. Ningxia Hui Autonomous Region, Wuzhong, Yanchi Country, on sandy ground under *Populusalba × P.berolinensis*, 22 Sep 2021, NX20210922-57 (FCAS3572), GenBank accession nos.: ITS-OM304279, LSU-OM304288, *rpb2*-OM421668; Shaanxi Province, Yulin, Dingbian Country, Yanchangbao Country, Xiliangwan Village, on sandy ground under *Populusalba × P.berolinensis*, 30 Sep 2021, SX20210930-65 (FCAS3573), GenBank accession nos.: ITS-OM304280, LSU-OM304289, *rpb2*-OM421669. Estonia. Saaremaa, Kaarma Municipality, Mändjala, open seashore forest with *Pinussylvestris* Linn., on fine calcareous sand, 19 Sep 2008, Jukka Vauras 26578F (TUR-A182630), GenBank no.: ITS and LSU-FJ904154.

### ﻿Muscarine detection

**Figure 4.**
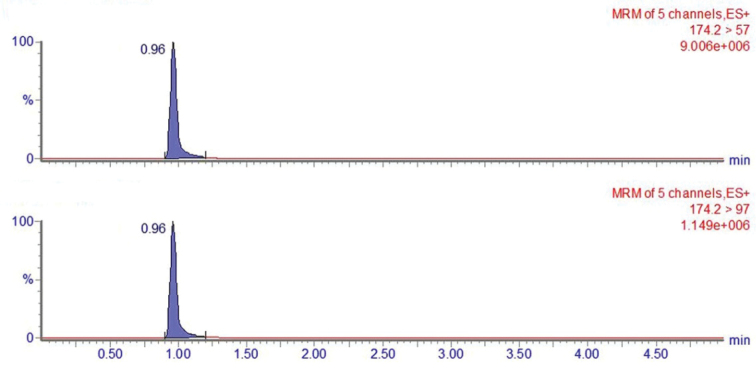
MRM chromatograms of muscarine from *Pseudospermaarenarium* (holotype).

The new species contained only muscarine (Fig. 4), whereas amatoxins, phallotoxins, ibotenic acid, muscimol, psilocybin and psilocin were not detected. The quantitative results are expressed by (X ± U; *k* = 2, *p* = 95%; Xu et al. 2020a, 2020b). The muscarine content in the holotype (NXYC20201005-01) between different basidiomata ranged from 3981.4 ± 796.4 to 4074.2 ± 801.3 mg/kg (*k* = 2, *p* = 95%) in the pilei and from 811.2 ± 162.3 to 883.3 ± 176.5 mg/kg (*k* = 2, *p* = 95%) in the stipes (Table 1). The muscarine content from different specimens ranged from 4012.2 ± 803.1 to 4302.3 ± 863.2 mg/kg (*k* = 2, *p* = 95%) in the pilei and from 850.4 ± 171.1 to 929.1 ± 184.2 mg/kg (*k* = 2, *p* = 95%) in the stipes (Table 2).

**Table 1.** Muscarine content in different parts from different basidiomata of the holotype (mg/kg).

**Collection number**	**Basidiomata 1**		**Basidiomata 2**		**Basidiomata 3**		**Basidiomata 4**		**Basidiomata 5**	
	**Stipe**	**Pileus**	**Stipe**	**Pileus**	**Stipe**	**Pileus**	**Stipe**	**Pileus**	**Stipe**	**Pileus**
NXYC20201005-01	816.2 ± 163.1	3981.4 ± 796.4	816.8 ± 17.1	4004.4 ± 801.1	811.2 ± 162.3	4025.4 ± 805.3	834.3 ± 167.1	4054.3 ± 801.6	883.3 ± 176.5	4074.2 ± 801.3

**Table 2.** Muscarine content in different parts from different specimens collected from different locations.

Collection numbers	Muscarine (mg/kg)	
	Stipes	Pilei
NXYC20201005-01	850.4 ± 171.1	4012.2 ± 803.1
NX20210922-57	929.1 ± 184.2	4302.3 ± 863.2
SX20210930-05	863.2 ± 172.5	4085.2 ± 816.2

## ﻿Discussion

The new species is known from three localities in Ningxia and Shaanxi of north-western China and is a locally common mushroom that occurs in late autumn under sandy poplar plantations (Fig. 2). As *Populusalba* × *P.berolinensis* plantations are widely distributed over north-western China, the new species may have broader distribution in adjacent areas. Moreover, the European material JV26578 collected in Estonia clustered with the new species with full support in the phylogenetic results. This collection also grew on calcareous fine sand, but under *Pinussylvestris*. According to the file notes, the specimen also has robust basidiomata (pileus up to 38 mm broad, stipes 50–55 × 7–9 mm) with ochraceous pileus and stipes and fungoid or slightly spermatic odour. The microfeatures of JV26578 have cylindrical-ellipsoid basidiospores measuring (13.5–)13.9–16.5(–18.2) × (7.1–)7.2–8.5(–8.8) μm (average, 15.3 × 7.7 μm), Q = 1.8–2.2(–2.25) [average, 1.98 (n = 20)] and clavate cheilocystidia measuring (34–)40–62(–70) × 14–20(–22) μm [average, 50 × 18 μm (n = 20)]. Except for the ochraceous tinge in pileus and stipes, no distinct macroscopical difference was observed between the Chinese materials and the Estonian specimen. The European specimen JV26578 is now considered conspecific with the Chinese materials. Accordingly, the new species has a Eurasian distribution.

*Pseudospermaarenarium* is characterised by its tricholomoid habit, dirty whitish to ochraceous and glabrous pileus, crowded lamellae with fimbriate edges, large cylindrical basidiospores and thin-walled cheilocystidia. The thick and long persistent velipellis gives its pileus a nearly smooth and whitish appearance. In the field, the pileus, stipe and lamellae surfaces are usually covered with humose sands, showing a dirty yellowish or sometimes brownish colour, especially in older individuals. Its mostly large cylindrical basidiospores are microscopically impressive, but cylindrical ellipsoid to elongated ellipsoid basidiospores also exist in the same individual. With the combination of the characteristics listed above, the new species is distinctive. Without examining its microscopic features or molecular sequence analyses, a mycologist or even an Inocybaceae specialist is unlikely to be able to identify it exactly into the genus *Pseudosperma*. Unexpectedly, the three-gene phylogeny places *P.arenarium* in the *P.rimosum* complex, which clusters with the lineage that unified the type material of *P.aureocitrinum* and a sample labelled as ‘P.cf.rimosum.’ However, *P.aureocitrinum* has a typical inocyboid habit, yellowish-tinged basidiomata and broadly ellipsoid to subovoid basidiospores and occurs in Mediterranean evergreen oak forests (Esteve-Raventós 2014).

*Pseudospermaarenicola* (R. Heim) Matheny & Esteve-Rav., a European species also occurring on coastal sandy soils, is similar in having a whitish appearance, long-persisting thick velipellis and long basidiospores, but it has a less robust habit, relatively short basidiospores measuring 11.5–12–18.5 × 6.0–6.4–7.5 μm (Kuyper 1986), different ecological associations and different phylogenetic positions (Heim 1931; Kuyper 1986; Stangl 1989). *Pseudospermapseudo-orbatum* (Esteve-Rav. & García Blanco) Matheny & Esteve-Rav. is a whitish species originally described from Spain, resembling the new species in having thick velipellis, non-rimose pileus, large cylindrical basidiospores and clavate cheilocystidia. However, it is distinguished by having pinkish lamellae when young, stockier stipes and an association with *Pinuspinaster* Ait. and *P.pinea* L. (Esteve-Raventós et al. 2003). *Pseudospermaniveivelatum* (D.E. Stuntz ex Kropp, Matheny & L.J. Hutchison) Matheny & Esteve-Rav., a North American species, shares white thick velipellis, elongated large basidiospores and ecological association with *Populustremuloides* Michx. and conifers. However, it differs by its sericeous pileus, shorter basidiospores measuring 11.5–13.9–18.5 × 6.0–6.4–7.5 μm, slender cheilocystidia (Kropp et al. 2013) and a clinically insignificant amount of muscarine (Kosentka et al. 2013).

Muscarine is a neurotoxin that causes salivation, sweating, delirium and even coma or death (Işiloğlu et al. 2009; Xu et al. 2020b). In recent years, more and more poisoning cases have been caused by eating Inocybaceae mushrooms containing toxic muscarine (Li et al. 2020, 2021a, 2022; Xu et al. 2020b). According to literature, five *Pseudosperma* species have been assayed; of these, *P.rimosum* (Bull.) Matheny & Esteve-Rav., *P.niveivelatum*, *P.sororium* (Kauffman) Matheny & Esteve-Rav. and *P.spurium* (Jacobsson & E. Larss.) Matheny & Esteve-Rav. contain muscarine; only *P.perlatum* (Cooke) Matheny & Esteve-Rav. was reported lacking muscarine (Kosentka et al. 2013). Xu et al. (2020b) reported that the muscarine content of *I.serotina* Peck in a poisoning incident was 324.0 ± 62.4 mg/kg wet weight. Li et al. (2021b) reported that the muscarine content in *I.squarrosolutea* (Corner & E. Horak) Garrido and *I.squarrosofulva* S.N. Li, Y.G. Fan & Z.H. Chen were 136.4 ± 25.4 to 1683.0 ± 313.0 and 31.2 ± 5.8 to 101.8 ± 18.9 mg/kg dry weight, respectively. Deng et al. (2021b, 2022) found that muscarine content in *Inospermamuscarium* Y.G. Fan, L.S. Deng, W.J. Yu & N.K. Zeng, *I.hainanense* Y.G. Fan, L.S. Deng, W.J. Yu & N.K. Zeng and *I.zonativeliferum* Y.G. Fan, H.J. Li, F. Xu, L.S. Deng & W.J. Yu were 16.03 ± 1.23, 11.87 ± 3.02 and [2.08 ± 0.05 (pileus) and 6.53 ± 1.88 (stipes)] g/kg dry weight, respectively. In this study, results showed that *P.arenarium* is a muscarine-positive species with middle- and upper-level muscarine content and also led to three poisoning incidents with a total of seven patients in northwest China during the past 2 years (Li et al. 2021a, 2022). Interestingly, the muscarine content in caps was approximately five times higher than in stipes. Although some studies showed that the toxin amount in the cap is higher than in the stipes (Hu et al. 2012; Garcia et al. 2015; Sun et al. 2018, 2019), the mechanism of such difference is still not clear. Li et al. (2021b) reported that the muscarine content of *Inocybesquarrosolutea* varied a lot in different specimens. However, in this study, the muscarine content in the mushroom samples collected from the same or three different places showed no significant difference. Additionally, no amatoxins, phallotoxins, ibotenic acid, muscimol, psilocybin and psilocin were detected in all samples. This study described *P.arenarium* as a new species, based on morphological, ecological, molecular and toxic evidence. The publicity and education of the new species are needed to control and prevent mushroom poisoning incidents.

## Supplementary Material

XML Treatment for Pseudosperma arenarium

## Supplementary materials

Supplementary material 1 Table S1 Data type Table (pdf file) Explanation note Information of taxa used in phylogenetic analysis. Newly sequenced collections are bold. https://binary.pensoft.net/file/736750This dataset is made available under the Open Database License (http://opendatacommons.org/licenses/odbl/1.0/). The Open Database License (ODbL) is a license agreement intended to allow users to freely share, modify, and use this Dataset while maintaining this same freedom for others, provided that the original source and author(s) are credited.Ya-Ya Yan ,Yi-Zhe Zhang, Jukka Vauras, Li-Na Zhao, Yu-Guang Fan, Hai-Jiao Li, Fei Xu
