# Supplementary material for: ﻿ Pseudospermaarenarium (Inocybaceae), a new poisonous species from Eurasia, based on morphological, ecological, molecular and biochemical evidence
Source: MycoKeys. 2022 Aug 30;92:79–93. doi: 10.3897/mycokeys.92.86277 (PMC9849055; doi:10.3897/mycokeys.92.86277)
Supplement: Supplementary material 1 — Table S1 [file mycokeys-92-079-s001.pdf]

Supplementary Table S1 Information of taxa used in phylogenetic analysis. Newly sequenced collections are in bold.

Taxa	Collection number (Herbarium)	Locality	Host/Habitat	GenBank accession number		
				ITS	LSU	RPB2
Pseudosperma aestivum	BK18089706 (UTC, holotype)	USA: Utah	*Populus*, *Abies*, *Pinus*, mid-elev	EU600847	EU600846	—
Pseudosperma albobrunneum	LAH35047 (holotype)	Pakistan	*Cedrus deodara*	MG495392	—	—
Pseudosperma amabile	BAN3010	Austria		MW010036	—	—
Pseudosperma amabile	BAN369 (holotype)	Germany		MW010031	—	—
***Pseudosperma arenarium* sp. nov.**	**NXYC20201005-01 (holotype)**	**China**	***Populus* plantations**	**OM304278**	**OM304287**	**OM421667**
***Pseudosperma arenarium* sp. nov.**	**NX20210922-57**	**China**	***Populus* plantations**	**OM304279**	**OM304288**	**OM421668**
***Pseudosperma arenarium* sp. nov.**	**SX20210930-65**	**China**	***Populus* plantations**	**OM304280**	**OM304289**	**OM421669**
Pseudosperma arenicola	RC-GB99-014	France	*Pinus*, *Salix*, sand dune	FJ904134	FJ904134	—
Pseudosperma arenicola var. *mediterraneum*	JV14920F (WTU)	Italy	*Pinus*, sandy seashore	JQ408748	JQ408748	—
Pseudosperma aureocitrinum	BAN2903 (isotype)	Spain		MW010047	—	—
Pseudosperma breviterincarnatum	BK28080407	USA: Utah		EU555451	EU555451	—
Pseudosperma breviterincarnatum	PBM1914	USA: Washington	sandy soil under *Abies*, *Picea*, *Pinus*	JQ408750	JQ319677	JQ846465
Pseudosperma brunneicothurnata	PBM1889 (TENN)	USA: Texas	*Fagus*, *Quercus*, *Pinus*	JQ408787	JQ319707	JQ846493
Pseudosperma brunneoumbonatum	MSM0053 (holotype)	Pakistan	*Pinus roxburghii*	MG742419	MG742420	—
Pseudosperma brunneoumbonatum	MSM00545	Pakistan	*Pinus roxburghii*	MG742421	MG742422	—
Pseudosperma bulbosissimum	EL6605	Norway	*Salix*, alpine	AM882765	AM882765	—
Pseudosperma bulbosissimum	EL75-07	Sweden		FJ904160	FJ904160	—
Pseudosperma cercocarpi	BK20069806 (UTC)	USA: Utah	*Cercocarpus-Pinus*, woodland	—	EU600890	EU600889
Pseudosperma citrinostipes	FYG2903	China		MT072897	MT071202	—
Pseudosperma citrinostipes	FYG2909 (holotype)	China		MT072898	MT071203	—

Pseudosperma conviviale	19793 (holotype)	Italy	*Quercus ilex*	MT095091	MT095115	—
Pseudosperma dulcamaroides	EL29-08	USA	*Salix*, alpine	FJ904127	—	—
Pseudosperma emberizanum	STU:SMNS-STU-F-0901461	Germany		MW647630	—	—
Pseudosperma fissuratum	PBM2195 (PERTH) (E7042)	Western Australia	*Eucalyptus*, tall wet forest	JQ408771	EU555466	EU555465
Pseudosperma fissuratum	PBM2206 PERTH (E7054) (holotype)	Western Australia	*Eucalyptus*, lawn	JQ408770	AY732213	JQ421069
Pseudosperma flavellum	EL13705	Sweden		AM882776	—	—
Pseudosperma cf. *flavellum*	EL 2010a / PAM05062502	France		FJ904128	FJ904128	—
Pseudosperma flavorimosum	LAH35042 (holotype)	Pakistan	*Pinus wallichiana A.B*	MG495391	—	—
Pseudosperma friabile	TENN068384 (type)	USA	In lawn under planted *Quercus phellos*	MH216095	NG_067823	—
Pseudosperma griseorubidum	CAL 1253	India: Kerala	*Vateria indica*	KT180326	KT180327	KT180328
Pseudosperma huginii	STU:SMNS-STU-F-0901564 (holotype)	Austria		NR_173974	MW647628	—
Pseudosperma hygrophorus	EL97-06	Sweden	*Betula*, *Salix*, subalpine meadow	FJ904137	FJ904137	—
Pseudosperma illudens	65726	Australia	natural bush under *Eucalyptus*	NR_153126	NG_057200	JQ421068
Pseudosperma keralense	KM 191712	India: Kerala	*Vateria indica*, *Hopea ponga*	KM924523	KM924518	KY553243
Pseudosperma lepidotellum	MCA1881 (BRG, holotype)	Guyana	In humus layer, on trunk of *Dicymbe*, rainforest	JN642233	JN642235	—
Pseudosperma melleum	MCVE 30145 (holotype)	Italy	*Quercus ilex and Quercus suber*	MT095090	MT095114	—
Pseudosperma melliolens	EL224-06	France	*Salix*, *Quercus*	FJ904149	FJ904149	—
Pseudosperma melliolens	PAM05052303	France	*Tilia*, calcareous soil	FJ904148	FJ904148	—
Pseudosperma melliolens	17130 (type)	France		MN901256	MN901256	—
Pseudosperma melliolens	MCVE30344	Italy	*Quercus ilex* & *Arbutus unedo*	MT095095	—	—
Pseudosperma cf. *microfastigiatum*	EL113-06	Sweden	*Dryas*	FJ904156	FJ904156	—
Pseudosperma cf. *microfastigiatum*	FO46800	Germany		DQ071697	DQ071697	—

Pseudosperma napaeanum	BAN2947 (holotype)	Germany		MW010040	—	—
Pseudosperma napaeanum	BAN2948	Germany		MW010044	—	—
Pseudosperma neglectum	ZT13022_DED8063 (SFSU)	Thailand	Lithocarpus and Castanopsis	EU600829	EU600829	—
Pseudosperma neoumbrinellum	HMJAU25742 (holotype)	China	*Populus*, *Salix*	MH047249	MG844977	—
Pseudosperma niveivelatum	PBM2337 (WTU)	USA: Washington	*Abies*, *Pinus*, sandy soil, montane	JQ313566	JQ313566	AY333776
Pseudosperma niveivelatum	BK21089714	USA	montane conifer-aspen forest (Pinaceae, *Populus*)	JQ319695	JQ319695	—
Pseudosperma obsoletum	EL1704	Sweden	*Picea*, *Corylus*	AM882769	AM882769	—
Pseudosperma obsoletum	PBM2332 (WTU)	USA	*Alnus*, *Corylus*	JQ408766	JQ408766	—
Pseudosperma occidentalis	BK27089703 (UTC, holotype)	USA: Utah	*Picea*, *Abies*, montane	EU600893	EU600893	EU600892
Pseudosperma occidentalis	PBM525	USA, Washington		AY038321	AY038321	AY333775
Pseudosperma perlatum	EL7404	Sweden	*Corylus*, *Betula*, calcareous soil	AM882771	AM882771	—
Pseudosperma perlatum	JV10247 (WTU)	Finland	*Populus tremula* mixed with *Betula*, *Alnus*, *Picea*, *Prunus*	JQ408767	JQ319698	—
Pseudosperma pinophilum	MSM0046 (holotype)	Pakistan	*Pinus roxburghii*	MG742414	MG742418	—
Pseudosperma pinophilum	MSM0047	Pakistan	*Pinus wallichiana*	MG742417	MG742415	—
Pseudosperma pomderosum	19060 (holotype)	Italy	*Quercus rubra*	MT095092	MT095116	—
Pseudosperma rimosum	AO20080250	GB		FJ904147	FJ904147	—
Pseudosperma rimosum	EL118-08	Sweden		FJ904146	FJ904146	—
Pseudosperma rimosum	EL211-06	France		FJ904145	FJ904145	—
Pseudosperma rimosum	PAM06112703	France: Corsica		FJ904143	FJ904143	—
Pseudosperma rimosum	EL75-05	Sweden	*Fagus*, *Quercus*, park	AM882762	AM882762	—
Pseudosperma rimosum	PAM03110904	France	*Quercus*	FJ904144	FJ904144	—
Pseudosperma rimosum	PBM2958	USA	*Quercus*, *Carya*, *Tsuga*, *Pinus*	JQ408777	JQ421071	JQ421071
Pseudosperma rimosum	DJL_SJ14 (TENN)	Virgin Islands	Coccoloba, Neea, dry tropical forest	JQ408784	EU600851	—

Pseudosperma rimosum	BK28080513 (UTC)	USA: Utah	*Populus, Pinus*	EU600850	EU600848	EU600849
Pseudosperma rimosum	PBM2601 (TENN)	Tennessee	*Betula*, *Picea*,	EU600852	EU600852	—
Pseudosperma cf. *rimosum*	PC080925	GB	*Pinus*, *Quercus*	FJ904153	FJ904153	—
Pseudosperma cf. *rimosum*	PAM05061101	France	*Tilia,* calcareous soil	FJ904155	FJ904155	—
Pseudosperma cf. *rimosum*	PBM2574 (TENN)	USA: Massachusetts	*Quercus*, *Pinus*	JQ408776	EF561633	EU307858
Pseudosperma cf. *rimosum*	JV8125	Finland	*Picea*, *Tilia*, *Populus*, rich	FJ904152	FJ904152	—
Pseudosperma cf. *rimosum*	JV22619	Estonia	*Quercus*, *Corylus*, calcareous soil	FJ904157	FJ904157	—
Pseudosperma cf. *rimosum*.	EL-2010d/JV26578	Sweden		FJ904154	FJ904154	—
Pseudosperma salentinum	MCVE 30342	Italy	*Quercus ithaburensis* subsp. *macrolepis*	MT095093	MT095117	—
Pseudosperma solare	BAN3078 (holotype)	Germany		NR_173973	MW647627	—
Pseudosperma sororium	MCA859/PBM3901 (TENN) (type)	USA: Virginia	*Quercus*	JQ408772	JQ319700	MH249810
Pseudosperma aff. *sororium*	PBM2654 (TENN)	USA: Tennessee	*Tsuga*, *Quercus*, *Carya*	EU600853	EU600853	—
Pseudosperma aff. *sororium*	ADW0057/TENN063512	USA	*Abies*, *Picea*	JQ408778	—	—
Pseudosperma aff. *sororium*	PBM3055/TENN063504	USA	redwood forest under *Pseudotsuga*, *Lithocarpus*	JQ408781	—	—
Pseudosperma aff. *sororium*	JV15200	Sweden	*Salix*, alpine	FJ904151	FJ904151	—
Pseudosperma aff. *sororium*	REH8245 (NY)	Costa Rica	*Quercus*, *Comarostaphylis*	JQ408783	JN975004	JQ421076
Pseudosperma squamatum	PAM05052301	France		FJ904132	FJ904132	—
Pseudosperma squamatum	SJ08003	Sweden	*Betula*, *Pinus*	FJ904136	FJ904136	—
Pseudosperma triaciculare	MSM0039 (holotype)	Pakistan	*Pinus roxburghii*	MG742423	MG742424	—
Pseudosperma triaciculare	MSM0041	Pakistan	*Pinus roxburghii*	MG742429	MG742430	—
Pseudosperma umbrinellum	JV13699	Finland	*Pinus*, *Populus*, *Salix*	FJ904165	FJ904165	—
Pseudosperma umbrinellum	PAM01102912	France		—	FJ904162	—

Pseudosperma umbrinellum	F14488 (type)	Italy		HM209796	–	–
Pseudosperma umbrinellum	PBM3024	USA	*Tsuga*, *Pinus*	MH216105	JN975010	JQ846497
Pseudosperma xanthocephala	PAM00100606	France	*Salix*	FJ904130	FJ904130	–
Pseudosperma yunnanense	HMJAU25840 (holotype)	China	*Quercus*, *Pinus*	MH047250	MG844975	–
Pseudosperma sp.	TR104-05 (M)	Papua New Guinea	Mixed *Castanopsis* forest, tropical montane	JN975011	JN975011	–
Pseudosperma sp.	TR133-05 (M)	Papua New Guinea	Mixed *Castanopsis* forest, tropical montane	JQ408791	JQ319709	–
Pseudosperma sp.	TR183-05	Papua New Guinea	Mixed *Castanopsis* forest, tropical montane	JQ408773	JN975005	–
Pseudosperma sp.	TR75-05	Papua New Guinea	Mixed *Castanopsis* forest, tropical montane	JQ408774	JQ815425	–
Pseudosperma sp.	TR138-05 (M)	Papua New Guinea	Mixed *Castanopsis* forest, tropical montane	JQ408792	JN975009	–
Pseudosperma sp.	TR49-05 (M)	Papua New Guinea	Mixed *Castanopsis* forest, tropical montane	JQ408790	JN975014	JQ421079
Pseudosperma sp.	MTS2494 (UC)	USA: California	*Quercus agrifolia*	JQ408786	JN975008	–
Pseudosperma sp.	BAN2931	Germany		MW010038	–	–
Pseudosperma sp.	BAN3013	Germany		MW010037	–	–
Pseudosperma sp.	MCA704 (TENN)	Japan	*Pinus*, *Betula*, montane	JQ408765	JN975007	–
Pseudosperma sp.	TM02-130	Ontario	*Tsuga* dominant forest plot	EU522733	EU 522733	–
Mallocybe terrigena	JV16431	Finland	*Pinus*, *Picea*, calcareous soil	AM882864	AY380401	AY333309
Nothocybe distincta	CAL-1310 (holotype)	India: Kerala		KX171343	EU604546	KX171345
